# Mispair-bound human MutS–MutL complex triggers DNA incisions and activates mismatch repair

**DOI:** 10.1038/s41422-021-00468-y

**Published:** 2021-01-28

**Authors:** Janice Ortega, Grace Sanghee Lee, Liya Gu, Wei Yang, Guo-Min Li

**Affiliations:** 1 Department of Radiation Oncology, University of Texas Southwestern Medical Center, Dallas, TX USA; 2 Department of Toxicology and Cancer Biology, University of Kentucky College of Medicine, Lexington, KY USA; 3 Laboratory of Molecular Biology, National Institute of Diabetes and Digestive and Kidney Diseases, National Institutes of Health, Bethesda, MD USA; 4 Present Address: Division of Viral Hepatitis, National Center for HIV/AIDS, Viral Hepatitis, STD and TB Prevention, Centers for Disease Control and Prevention, Atlanta, GA USA

**Keywords:** Molecular biology

## Abstract

DNA mismatch repair (MMR) relies on MutS and MutL ATPases for mismatch recognition and strand-specific nuclease recruitment to remove mispaired bases in daughter strands. However, whether the MutS–MutL complex coordinates MMR by ATP-dependent sliding on DNA or protein–protein interactions between the mismatch and strand discrimination signal is ambiguous. Using functional MMR assays and systems preventing proteins from sliding, we show that sliding of human MutSα is required not for MMR initiation, but for final mismatch removal. MutSα recruits MutLα to form a mismatch-bound complex, which initiates MMR by nicking the daughter strand 5′ to the mismatch. Exonuclease 1 (Exo1) is then recruited to the nick and conducts 5′ → 3′ excision. ATP-dependent MutSα dissociation from the mismatch is necessary for Exo1 to remove the mispaired base when the excision reaches the mismatch. Therefore, our study has resolved a long-standing puzzle, and provided new insights into the mechanism of MMR initiation and mispair removal.

## Introduction

The highly conserved DNA mismatch repair (MMR) system maintains genome stability primarily by correcting DNA replication errors in the newly synthesized strands. Defects in MMR cause hereditary and sporadic cancers.^1–5^ Recent studies have also shown that tumors’ MMR activity predominantly modulates checkpoint blockade immunotherapy.^6–9^ Thus, understanding the molecular mechanism of MMR is important for predicting cancer susceptibility and designing effective therapies. The MMR process can be divided into three steps: (1) mismatch recognition by MutS family proteins, specifically MutS in prokaryotes and MutSα (MSH2·MSH6 heterodimer) or MutSβ (MSH2·MSH3) in eukaryotes; (2) mismatch removal by an exonuclease, e.g., exonuclease 1 (Exo1) in eukaryotes in a manner that depends on MutS proteins, MutL family proteins (MutL in prokaryotes and MutLα made of MLH1·PMS2 in mammals), RFC (replication factor C), and PCNA (proliferating cellular nuclear antigen); and (3) DNA re-synthesis by a replicative DNA polymerase assisted by replication factors RFC, PCNA, and RPA (replication protein A).^1–4^

Unlike DNA lesions of modified bases, abasic sites, or strand breaks, which contain obvious abnormalities, mismatches such as a G–T pair and a small insertion-deletion loop consist of normal nucleotides that are mispaired. In addition, to ensure replication fidelity, the MMR process has to distinguish which base in a mismatch is incorrect. Remarkably, the MMR system knows to target the newly synthesized strand for mismatch removal and depends on strand discrimination signals, such as ssDNA breaks, in the daughter strand. However, an ssDNA break can be several hundred base pairs away from the mispaired base, and it is unclear how mismatch binding by a MutS protein triggers DNA excision at a strand break that is far away from the mismatch.

**Fig. 1:**
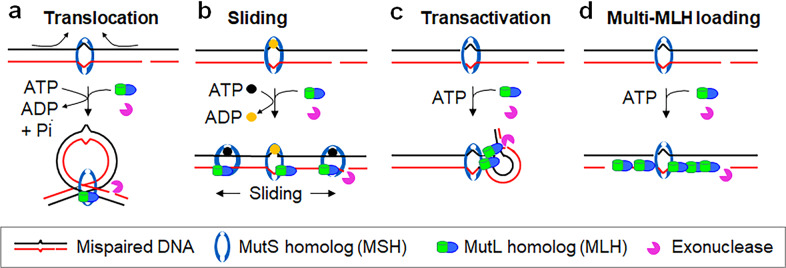
Current models of MMR initiation. **a** The translocation model suggests that an α-like loop structure forms as a result of the “bidirectional” translocation of MutS homologs when searching for the strand discrimination signal.^11^ **b** The molecular switch model postulates that MSH homologs bind to the mismatch and then slide away from the site to search for the strand discrimination signal in an ATP-dependent manner.^14^ **c** The transactivation model suggests that the MMR initiation complex remains bound to the mismatch and activates downstream nuclease activities at the strand signal via DNA bending/looping.^19,20,22^ **d** The multi-MLH loading model suggests that mismatch-bound MutS homologs recruit multiple molecules of MutL homologs flanking the mismatch.^21^

Several models (Fig. 1) have been proposed to answer this fundamental and long-standing question in MMR.^10^ The translocation and sliding models claim that MutS proteins (i.e., MutS, MutSα and MutSβ) initially bind to the mismatch, but subsequently move away from it upon ATP binding or hydrolysis to search for the strand discrimination signal.^11,12^ These models appear consistent with the fact that MutS proteins can slide along the DNA helix^13–17^ and that strand discontinuity inhibits MutH endonuclease activity at the hemimethylated GATC site.^18^ The transactivation and multi-MLH loading models suggest that MutS proteins stay bound to the mismatch during the communication between the mismatch and strand discrimination signal.^19–22^ Evidence supporting these models includes: (1) all crystal structure studies of MutS proteins, except one that shows limited DNA sliding of the MutS–MutL complex,^23^ do not identify a sliding transitional MutS–DNA complex;^24–27^ (2) mismatch binding by MutS on a heteroduplex DNA activates MutH cleavage of a hemimethylated GATC site located on a separate DNA molecule without a mismatch,^20^ which contradicts the study by Pluciennik et al.;^18^ (3) data from a yeast study directly visualizing MMR proteins during DNA replication do not support MutS sliding;^21^ (4) biotin-streptavidin blockades between a mismatch and a pre-existing nick do not block mismatch-provoked excision at the pre-existing nick 3′ to the mismatch in human nuclear extracts,^28^ but the work is concerned with whether or not the observed 3′ → 5′ excision is mismatch provoked, as such an exonuclease has not been identified for 3′-directed MMR in human cells.

Although the exact reason for this controversy remains to be clarified, using different kinds of DNA substrates, various reaction conditions, and incomplete reconstituted MMR systems may have contributed to the discrepancy. Nevertheless, the sliding of MutS proteins can be easily blocked by a physical barrier such as the LacI repressor protein^15^ and streptavidin,^12^ and traveling a relatively long distance along the DNA helix during replication by MutS proteins is challenging because the replication machinery and histone chaperone and assembly activities are tightly associated with the replication fork.^29^ Conversely, if MutS proteins remain bound at the mismatch, why do they have a sliding activity?

We performed functional assays of complete MMR using two different systems that inhibit MutS sliding to the strand break (or strand discrimination signal, where Exo1 acts). In one system, movement of the human MutSα and/or MutSα–MutLα complex was constrained within two LacI roadblocks; in the other system, sliding-deficient MutSα proteins were generated and used to conduct MMR. We show that sliding of MutSα is not required for initiating mismatch-provoked DNA excision at a distal nick, but essential for yielding the right of way to Exo1 for mismatch removal. We also found that regardless of 3′ nick-directed or 5′ nick-directed MMR, the first reaction is the endonucleolytic incision by MutLα, rather than exonucleolytic excision at the pre-existing strand break. In fact, it is the excision at an incision site 5′ and near the mismatch that leads to mismatch removal by Exo1. This study has resolved a long-standing controversy in MMR and provides novel insights into the mechanism of MMR initiation and mismatch removal.

## Results

### Protein “roadblocks” cannot completely inhibit in vitro MMR

**Fig. 2:**
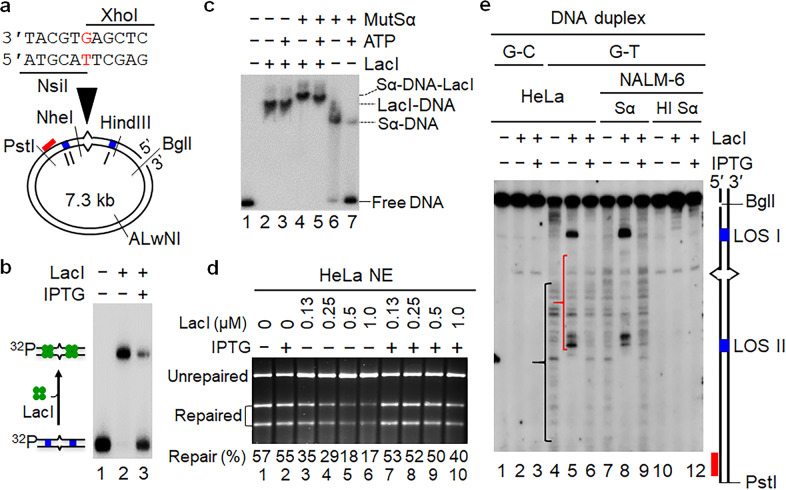
LacI roadblocks effectively block MutSα sliding. **a** The DNA heteroduplex used for in vitro MMR. The circular DNA substrate contains a 5′ nick at the *Bgl*I restriction site, a G–T mispair, and two LacI binding sites (blue bars) that are separated by 130 bp. The G–T mismatch was placed in the overlapping recognition sequences of *Nsi*I and *Xho*I, so that the heteroduplex is resistant to cleavage by both enzymes. The nick-directed MMR removes the mispaired base and subsequent DNA resynthesis restores the sensitivity of the repair product to *Nsi*I, which was used to score for repair. **b** EMSA assay to determine the specific interaction between LacI and LOS. **c** EMSA analysis to determine efficient blockage of MutSα sliding by the LacI roadblocks. **d** In vitro MMR assay showing partial inhibition of MMR in HeLa nuclear extract (NE) by LacI. **e** Southern blot analysis to determine mismatch-provoked excision intermediates with or without LacI. DNA fragment derived from the circular substrate by a *Bgl*I-*Pst*I double digestion contains (from top to bottom) the original strand break, first LacI operon sequence (LOS) I, mismatch, LOS II, and probe annealing site. The red bar represents the ^32^P-labeled oligonucleotide probe. Sα and HI Sα stand for MutSα and heat-inactivated MutSα, respectively. In all LacI-containing reactions, LacI was preincubated with DNA substrates on ice for 10 min before adding other reaction components.

The interaction between LacI and lactose operator has been well characterized and widely used to block movement of MutSα^15,30,31^ and the factors involved in DNA replication^32^ along the DNA helix. By exploiting the LacI–lac operator interaction system, we established an in vitro MMR system that reversibly blocks human MutSα sliding along the DNA helix. An oligonucleotide duplex containing the lac operator sequence (LOS) was cloned into the *Hind*III site (Fig. 2a, Site I) of M13mp18-UKY1 and M13mp18-UKY2,^33^ which already harbors a native LOS (Fig. 2a, Site II). The resulting M13mp18-UKY-Lac phage series were used to construct a G–T mismatched heteroduplex with a LOS at either side of the mismatch (Fig. 2).

To establish that the premise and assumptions of this experimental system are correct, a 282-bp duplex containing a mismatch and two LOS sites was generated (Fig. 2a) from the M13mp18-UKY-Lac phage DNAs (see Materials and Methods for details). The 282-bp duplex was examined for its interactions with LacI and human MutSα by electrophoretic mobility shift assay (EMSA) with or without Isopropyl β-D-1-thiogalactopyranoside (IPTG), an allolactose mimic that specifically interacts with and prevents LacI from binding to LOS. As expected, LacI efficiently bound to LOS, and IPTG released > 70% of the binding (Fig. 2b), confirming a specific but reversible interaction between LacI and LOS which is regulated by IPTG. We then examined the impact of the LacI “roadblock” system on MutSα sliding on mismatched DNA. The results showed that LacI binding does not interfere with MutSα binding to DNA, because MutSα supershifted the LacI-bound heteroduplex (Fig. 2c, lane 4). However, the presence of LacI altered MutSα’s response to ATP. Usually, ATP destabilized the MutSα–DNA complex^34^ (Fig. 2c, lane 7), but in the presence of LacI, ATP failed to dissociate MutSα from DNA (Fig. 2c, lane 5), and the DNA substrate remained similarly “supershifted” in reactions that contain both LacI and MutSα, regardless of the presence of ATP (Fig. 2c, lanes 4 and 5). These results indicate that as in the yeast system,^15^ the LacI–LOS interaction functions as an effective “roadblock” for human MutSα.

To test the impact of the LacI roadblocks on MMR, we performed in vitro functional MMR assays.^35^ A circular G–T heteroduplex (25 fmol or 1.25 nM) containing a LOS site on either side of the mismatch (Fig. 2a) was pre-treated with or without an increasing amount (10 nM–1 µM) of LacI tetramer before incubation with MMR-competent HeLa nuclear extracts. Surprisingly, we found that LacI does not inhibit in vitro MMR when the LacI:DNA ratio is < 30 (Supplementary information, Fig. S1). The repair activity was compromised only when the LacI:DNA ratio reached > 30 (Fig. 2d, lanes 3–6); LacI at a concentration that is 800-fold more than that of DNA substrates could not completely block MMR activity in HeLa nuclear extracts (Fig. 2d, lane 6). These results suggest that sliding of MutSα from the mismatch to the strand break is not required for MMR.

### Mismatch-provoked excision occurs when sliding of MutSα is blocked

To understand why LacI roadblocks can block MutSα sliding, but not MMR, we measured the excision intermediates associated with mismatch-provoked excision by conducting MMR assays using the same G–T heteroduplex (Fig. 2a) in the absence of exogenous dNTPs,^35^ which allows mismatch-provoked excision, but largely inhibits repair-associated DNA synthesis. Excision intermediates were detected by a ^32^P-labeled probe (red bar) that is complementary to the nicked strand near the *Pst*I site after restriction digestions (*Pst*I and *Bgl*I) and gel electrophoresis. The results of this analysis are shown in Fig. 2e. As expected, HeLa nuclear extracts can efficiently catalyze mismatch-provoked excision in the absence of LacI (Fig. 2e, lane 4), with most excision products (indicated by a black bracket) mapped downstream of the mismatch site. However, in the presence of LacI (250 nM), two dominant excision products were observed (Fig. 2e, lane 5), one above and the other below the mismatch marker. Interestingly, both products were mapped right at the boundary of the LOS sites (see blue boxes), suggesting that the LacI roadblock also blocks the excision path. The product mapped at LOS I was obviously derived from the excision that started at the pre-existing strand break (the *Bgl*I site) and ended at LOS I by LacI. Because these molecules are between the mismatch and the strand break, they still contain the mismatch, and likely represent the partially inhibited repair by LacI. However, we also observed abundant excision/incision intermediates between the mismatch and LOS II (see the red bracket in lane 5). The relative location of these molecules, i.e., downstream of the mismatch, suggests that the mispaired base has been removed in these molecules. Because the excision from the pre-existing strand break is blocked by LacI, mismatch removal in these molecules must result from initial incision, followed by excision. In other words, incisions between LOS I and the mismatch and subsequent excision by Exo1 have occurred, contributing to the observed repair in reactions with high concentrations of LacI (Fig. 2d, lanes 3–6). This explains why LacI roadblocks cannot completely inhibit MMR. Taken together, these results indicate that even though sliding of MutSα is constrained within two LacI roadblocks, mismatch-provoked excision and incision occur outside and inside the roadblocks, respectively, i.e., sliding of MutSα is not involved in mismatch-provoked excision/incision.

To ensure that the excision/incision products accumulated at LOS I and LOS II were generated in a mismatch-provoked and MMR-specific manner, in vitro excision assays were performed using a homoduplex DNA substrate (G–C) and nuclear extract derived from an *MSH2*-deficient cancer cell line, NALM-6.^36^ Our results show that regardless of the presence of LacI and/or IPTG, excision intermediates were rarely detected in HeLa extract reactions when the homoduplex DNA substrate was used (Fig. 2e, lanes 1–3), indicating that excision intermediates observed in reactions with the heteroduplex (Fig. 2e, lanes 4–6) are provoked by the mismatch. The results from the *MSH2*-deficent NALM-6 nuclear extract show that only the reaction supplemented with active MutSα, but not heat-inactivated (HI) MutSα, yielded excision/incision products (Fig. 2e, lanes 7–9), implying that generation of these repair intermediates depends on a functional MMR system. Collectively, these results further indicate that mismatch-provoked excision/incision occurs even though MutSα is constrained within the LacI roadblocks.

### Sliding-deficient MutSα triggers mismatch-provoked excision

**Fig. 3:**
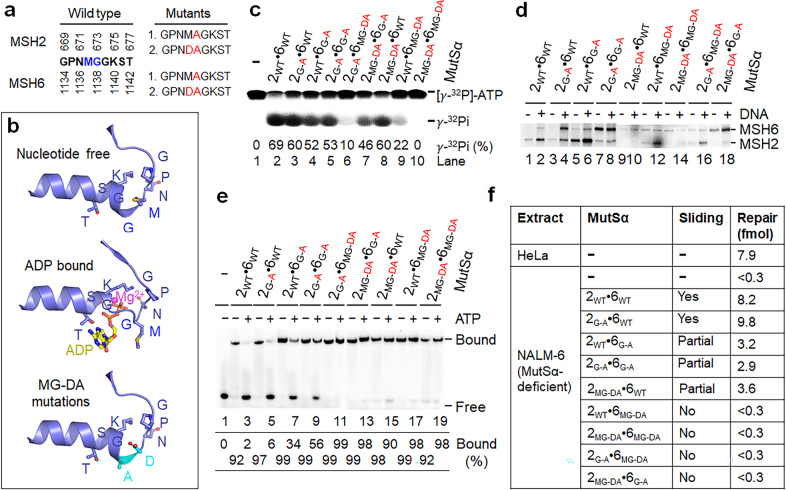
Sliding-deficient MutSα is defective in MMR. **a** Amino acid sequences and positions of the Walker A motif in the MSH2 and MSH6 subunits of MutSα. Mutagenesis was focused on methionine (M) and glycine (G) (in blue) in the WT sequence, with the corresponding mutated residues in red. **b** Structures of Walker A motif of MSH6. Nucleotide-free (upper panel) and ADP-bound (middle panel) MSH6 structures (PDB: 2O8E and 2O8B) are shown in the blue cartoon. The conserved residues and ADP are depicted as sticks and balls and labeled. MG to DA mutations (highlighted in cyan, bottom panel) stabilize the helical conformation, thus making the protein resistant to nucleotide binding. **c** ATPase activities of WT and mutant MutSα proteins. **d** ATP-binding activity of WT and mutant MutSα proteins with or without mismatched DNA. Proteins were incubated with [γ-^32^P]ATP, followed by UV cross-linking and SDS-PAGE. **e** EMSA analysis to determine ATP-dependent dissociation of WT and mutant MutSα proteins from a 36-bp heteroduplex DNA. **f** In vitro MMR assay to determine the ability of individual MutSα proteins to restore MMR in MutSα-deficient nuclear extract.

Next, we tested the involvement of MutSα’s sliding activity in mismatch-provoked excision using sliding-deficient MutSα proteins. We propose that if the sliding activity is not essential for MMR initiation, MutSα that is defective in sliding but retains other MMR-related functions, including mismatch binding, should be able to initiate an MMR reaction. It has been established that MutS sliding is ATP-binding dependent.^13,30,37^ The intact Walker A motif (GPNMGGKST) of MutS family proteins, including MSH2 and MSH6 (Fig. 3a), is necessary for ATP/ADP binding. We therefore focused our mutagenesis efforts outside of the core Walker A motif (GKST), which directly interacts with the ATP/ADP nucleotide. The MG conserved among human MSHs before GKST undergo conformational changes from α-helical to a loop structure upon ATP or ADP binding (Fig. 3b).^26^ By replacing MG with DA (Fig. 3b, bottom panel), which would stabilize the α-helix structure because Ala (A) has higher helical forming propensity than the Gly and Asp (D) N-caps helix by charge–charge interactions (Fig. 3b), mutant MutS proteins are predicted to have reduced ATP-binding activity. We constructed MutSα mutants with alanine substitutions at G_673_ of MSH2 (MSH2_G-A_ or 2_G-A_) and G_1138_ of MSH6 (MSH6_G-A_ or 6_G-A_), aspartic acid (D) and alanine (A) double substitutions at M_672_G_673_ of MSH2 (MSH2_MG-DA_ or 2_MG-DA_) and M_1137_G_1138_ of MSH6 (MSH6_MG-DA_ or 6_MG-DA_). MutSα heterodimers carrying one or both mutant subunits were generated (Supplementary information, Fig. S2) and extensively characterized for their MMR-related activities.

Analysis of ATPase activity revealed that all mutant MutSα proteins exhibited lower ATPase activity than wild-type (WT) MutSα (Fig. 3c); the MutSα proteins containing a MSH6_MG-DA_ subunit displayed a dramatic reduction in ATP hydrolysis (Fig. 3c, lanes 6, 9 and 10), with MSH2_MG-DA_·MSH6_MG-DA_ being completely defective in ATPase activity (lane 10). ATP-binding experiments showed that MSH2_MG-DA_·MSH6_MG-DA_ exhibited little ATP-binding activity (Fig. 3d, lane 14). These observations are in agreement with the structure-based prediction. We showed that MG→DA mutations greatly reduced ATP binding and thus ATPase activity of MutSα.

To determine the impact of ADP or ATP binding on MutSα sliding activity, we performed EMSA (Fig. 3e). The results reveal that all MSH6_MG-DA_-containing MutSα proteins (lanes 11, 17 and 19), as well as MSH2_MG-DA_·MSH6_G-A_ (lane 13), showed little ATP-dependent sliding activity, while the other mutants partially lost their DNA sliding activity (lanes 5, 7, 9 and 15). Consistently, the MutSα mutants defective in DNA sliding (i.e., MSH2_WT_·MSH6_MG-DA_, MSH2_G-A_·MSH6_MG-DA_, MSH2_MG-DA_·MSH6_G-A_, MSH2_MG-DA_·MSH6_MG-DA_) also failed to restore MMR in MutSα-deficient extract, while those partially defective in DNA sliding (i.e., MSH2_WT_·MSH6_G-A_, MSH2_G-A_·MSH6_G-A_, MSH2_MG-DA_·MSH6_WT_) retained partial MMR activity (Fig. 3f), consistent with a previous yeast study that used other ATP binding-deficient MutSα mutants.^31^ These results indicate that MutSα’s DNA sliding activity is essential for MMR.

**Fig. 4:**
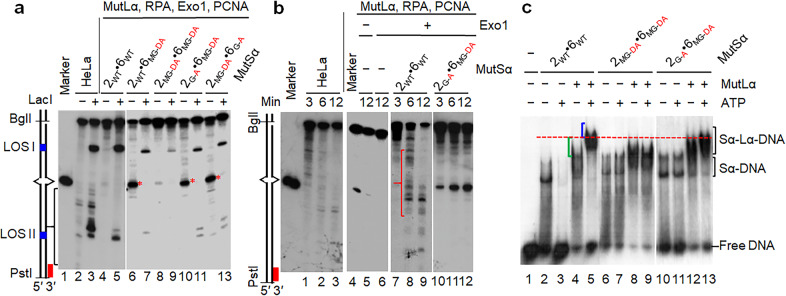
Sliding-deficient MutSα triggers mismatch-provoked excision but blocks the excision path at the mismatch site. **a** Southern blot analysis to determine mismatch-provoked excision conducted by sliding-deficient MutSα in the reconstituted MMR system with or without the LacI roadblocks, as indicated. The reaction mixtures were incubated at 37 °C for 20 min before being processed for Southern hybridization analysis. **b** Southern blot analysis to show accumulation of excision intermediates at the mismatch site in the reconstituted MMR system with sliding-deficient MutSα proteins over time. **c** EMSA analysis to show that the MMR initiation complex contains multiple molecules of MutLα. DNA substrate (0.1 pmol) was a ^32^P-labeled 100-bp duplex containing a G–T mismatch in the middle. The concentration of MutSα or MutLα used was 2 pmol.

We next asked whether sliding-deficient MutSα proteins block the initiation of mismatch-provoked excision at the pre-existing nick. To answer the question, we determined mismatch-provoked excision/incision in reconstituted MMR reactions that contained MutSα or a sliding-deficient MutSα (i.e., MSH2_WT_·MSH6_MG-DA_, MSH2_G-A_·MSH6_MG-DA_, MSH2_MG-DA_·MSH6_G-A_ or MSH2_MG-DA_·MSH6_MG-DA_) with or without LacI. If MutSα’s sliding from the mismatch to the nick is essential for Exo1 recruitment and activation, no excision would occur when MutSα stays bound to the mismatch. However, we found that in the presence of LacI, reconstituted reactions containing each of the sliding-deficient MutSα, except for the double mutant MSH2_MG-DA_·MSH6_MG-DA_, accumulated the same excision products at LOS I (Fig. 4a, lanes 7, 11, and 13) as those with WT MutSα (Fig. 4a, lane 5) or HeLa nuclear extracts (Fig. 4a, lane 3). These results reveal that although these sliding-deficient MutSα proteins are defective in MMR (Fig. 3f), they can trigger mismatch-provoked excision at a pre-existing nick, further supporting the notion that the DNA sliding activity of MutSα is not involved in initiating mismatch-provoked excision.

### Sliding-deficient MutSα blocks mismatch removal at the mismatch site

To elucidate the mechanism through which sliding-deficient MutSα proteins do not block mismatch-provoked excision at the pre-existing nick, but still inhibit MMR, we analyzed and compared the excision intermediates generated in reactions with WT and sliding-deficient MutSα proteins without the LacI roadblocks. As expected, no excision products were accumulated at the LOS I site in the MutSα-containing reaction when LacI was omitted (Fig. 4a, lane 4), and the excision then removed the mismatched base. The excision products downstream of LOS I were hardly detectable in this reaction (Fig. 4a, lane 4), probably because over-digestion by Exo1 during a 20-min incubation, led to destruction of the probe annealing site (red bar). This prediction was confirmed when the reaction time was reduced to 6 and 12 min (Fig. 4b, lanes 8 and 9, see red bracket). In reactions containing sliding-deficient MutSα proteins MSH2_WT_·MSH6_MG-DA_ (Fig. 4a, lane 6), MSH2_G-A_·MSH6_MG-DA_ (Fig. 4a, lane 10), and MSH2_MG-DA_·MSH6_G-A_ (Fig. 4a, lane 12), omitting LacI also eliminated the excision intermediates accumulated at the LOS I site; however, a new product (marked by a red asterisk) appeared right at the site of the mismatch (Fig. 4a, lanes 6, 10 and 12), which was almost invisible in the reaction with MutSα (Fig. 4a, lane 4). These observations suggest that the excision roadblock in these reactions are the mutant MutSα proteins themselves, as they failed to dissociate (Fig. 3e) from the mismatch when Exo1-catalyzed excision reaches the mismatch. This second roadblock also explains why only residual excision products were observed at LOS II in reactions with all sliding-deficient MutSα proteins in the presence of LacI (Fig. 4a, lanes 7, 9, 11 and 13), in comparison with reactions with WT MutSα (Fig. 4a, lanes 3 and 5). These results provide the molecular basis of why these sliding-deficient MutSα proteins can initiate mismatch-provoked excision at the pre-existing nick, but defective in overall MMR. We therefore have identified a novel biological function for the sliding activity of MutS family proteins, which is to yield the right of way to nucleases (e.g., Exo1 in human cells) to remove the mispaired base when the excision approaches the MutS-bound mismatch. Alternatively, the excision blockage at the mismatch site by these MutSα mutants could be due to their failure in ATP binding-induced conformational changes, which may prevent MutSα dissociation from DNA.

We observed that the MutSα mutant MSH2_MG-DA_·MSH6_MG-DA_ behaved differently from the ones described above, although all of them irreversibly bound to mismatched DNA regardless of the presence or absence of ATP (Fig. 3e). While other MutSα mutants promote excision intermediates at LOS I in the presence of LacI and cause excision termination at the mismatch site in the absence of LacI (Fig. 4a), the MSH2_MG-DA_·MSH6_MG-DA_ mutant could barely generate excision products at either LOS site under the same conditions (Fig. 4a, lanes 8 and 9). This correlates with more abundant full-length DNA substrates in these reactions (compare the top bands in lanes 8 and 9 with those in the other reactions). These results suggest that the MSH2_MG-DA_·MSH6_MG-DA_ heterodimer has lost its ability to trigger mismatch-provoked excision both outside and inside of the LacI roadblocks.

### The MSH2_MG-DA_·MSH6_MG-DA_ MutSα fails to form an active initiation complex with MutLα

MutL family proteins are known to be an initiation factor by interacting with MutS proteins to form an active initiation complex in an ATP-dependent manner.^11,12,19–21,38,39^ Because MSH2_MG-DA_·MSH6_MG-DA_ has completely lost its ATP-binding activity (Fig. 3d, lane 14), we postulate that the MSH2_MG-DA_·MSH6_MG-DA_ mutant fails to form an active initiation complex with MutLα. We therefore performed EMSA to determine MutLα’s interactions with WT and several mutant MutSα proteins on a 100-bp G–T mismatched DNA. In the absence of ATP, incubating MutLα with MutSα and the heteroduplex DNA produced a supershifted complex (indicated by a green bracket in Fig. 4c), which migrates more slowly than the MutSα–DNA complex (Fig. 4c, lane 4). This complex was also observed in reactions with mutant MutSα proteins MSH2_MG-DA_·MSH6_MG-DA_ (Fig. 4c, lane 8) and MSH2_G-A_·MSH6_MG-DA_ (Fig. 4c, lane 12) under the same condition, indicating the formation of a MutSα–MutLα–DNA complex. However, in the presence of ATP, a more slowly migrating MutSα–MutLα–DNA complex (see blue bracket) was detected in reactions containing WT MutSα (Fig. 4c, lane 5) and MSH2_G-A_·MSH6_MG-DA_ (Fig. 4c, lane 13), but not in the reaction containing MSH2_MG-DA_·MSH6_MG-DA_ (Fig. 4c, lane 9). These results reveal the following facts: (1) an active MutSα–MutLα complex contains more than one molecule of MutLα, and stays bound to DNA in the presence of ATP (Fig. 4c, lane 5), confirming again that the activated MMR initiation complex does not slide away from the mismatch during MMR initiation; (2) MSH2_MG-DA_·MSH6_MG-DA_ fails to form an active MutSα–MutLα initiation complex, which explains why this MutSα mutant is unable to trigger mismatch-provoked excision at the pre-existing nick and incision between two LOS sites (Fig. 4a, lanes 8 and 9). In comparison with other sliding-deficient MutSα proteins that can trigger mismatch-provoked excision and incision, MSH2_MG-DA_·MSH6_MG-DA_ lacks ATP-binding activity (Fig. 3b, d) that is essential for activating other mismatch repair proteins such as MutL family proteins.^20^ Nevertheless, some other ATP binding-deficient MutSα proteins that can interact with MutLα have also been reported.^31^

### MutLα cleaves the nicked DNA strand 5′ to the mismatch

**Fig. 5:**
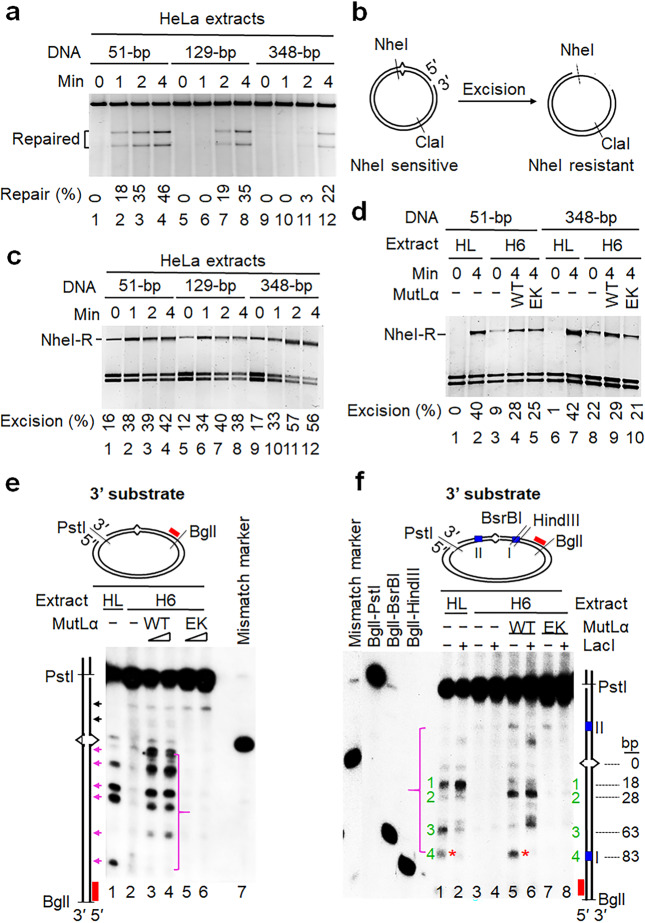
MutLα is essential for both 3′- and 5′-directed MMR by nicking the newly synthesized strand 5′ near the mismatch. **a** In vitro MMR assay in HeLa nuclear extracts shows that MMR efficiency is inversely proportional to the distance that separates mismatch and strand break. **b** Principle of mismatch removal assay. **c** Mismatch removal assay to show that mismatch removal efficiency is the same for all heteroduplexes with various distances between mismatch and strand break. **d** Mismatch removal assay to show that MutLα endonuclease activity is the determining factor for efficient mismatch removal in heteroduplexes with a long distance between mismatch and strand break. All reactions were incubated at 37 °C for 4 min. **e** Southern blot analysis shows that MutLα makes multiple incisions (indicated by pink arrows) 5′ to the mismatch, which can effectively remove the mispaired base. **f** Southern blot analysis shows that LacI roadblocks slightly alter the incision pattern of MutLα, but do not inhibit MutLα endonuclease activity. Green numbers show major incisions 5′ to the mismatch in HeLa nuclear extracts, and their estimated distances (bp) from mismatch are shown in right side of the gel. HL, HeLa nuclear extracts; H6, HCT116 nuclear extracts; EK, a MutLα mutant carrying an E705K substitution in the PMS2 subunit.

Our LacI roadblock results reveal that although the excision path at the pre-existing strand break is blocked at LOS I, multiple pieces of repair intermediates were detected between the mismatch and pre-existing strand break (Figs. 2 and 4). Therefore, we postulate that these intermediates are derived from endonucleolytic cleavage, but not exonucleolytic digestion. If this were true, mismatch removal would not depend on the excision all the way from the pre-existing break, but on the excision from an initiation-triggered nick immediately 5′ to the mismatch. To confirm this, we performed in vitro MMR assays using the G–T heteroduplex (Fig. 2a) containing a pre-existing strand break 51 bp, 129 bp, or 348 bp away from the mismatch, which were designated as 51-bp, 129-bp, or 348-bp heteroduplex, respectively. Results from the time course experiments show that the repair rate is inversely correlated with the distance between mismatch and strand break because the repair products can be detected as early as 1 min for the 51-bp substrate, but 2 min and 4 min for the 129-bp and the 348-bp substrates, respectively (Fig. 5a). The same is true for repair efficiency in these substrates (compare repair rates in lanes 4, 8, and 12), consistent with a previous study.^40^ These results indicate a delay in repairing heteroduplexes with a longer distance between the two sites. However, the delayed repair can be due to delayed removal of the mispaired base or delayed DNA synthesis because of distance-dependent signal communication required for activation of excision or repair synthesis.

To distinguish these possibilities, we performed mismatch removal assays. This assay exploits the presence of a unique *Nhe*I restriction site immediately (6-bp) 3′ to the mismatch (Fig. 5b), thus the *Nhe*I sequence is simultaneously removed along with the mispaired base during mismatch-provoked excision, which converts the *Nhe*I sequence to ssDNA and makes it resistant to *Nhe*I digestion.^33^ The results show no fundamental difference in the amount of *Nhe*I-resistant DNA generated in all three DNA heteroduplex reactions at any given time (Fig. 5c). This suggests that despite significant differences in distance between mismatch and pre-existing strand break, mismatch removal for all these DNA substrates is equally efficient. The observed delay in completing the repair for the substrates with a longer distance between mismatch and strand break is probably due to post-excision events. These results reveal that mismatch removal in all heteroduplexes may occur via initial endonuclease cleavage 5′ to and near the mismatch, followed by exonuclease digestion from the resulting nick.

Because MutLα is a strand-specific endonuclease,^41^ we suspect that efficient mismatch removal in heteroduplexes with a long distance between mismatch and pre-existing nick is enabled by a MutLα cleavage at a place 5′ to and near the heterology, followed by Exo1-catalyzed excision to remove the mispaired base. Therefore, we conducted the mismatch removal assay using an extract derived from MutLα-deficient colorectal cancer line HCT116 (H6)^42^ supplemented with purified MutLα. A background level of *Nhe*I-resistant products was observed in reactions with H6 extract alone for both 51-bp and 348-bp substrates (Fig. 5d, lanes 3 and 8); this could be due to the fact that in the absence of MutLα, which negatively regulates Exo1 nuclease activity,^9^ Exo1 spontaneously conducts limited excision during reaction assembly. However, adding WT MutLα to H6 extracts enhanced the production of the *Nhe*I-resistant species for both DNA substrates (Fig. 5d, lanes 4 and 9). Interestingly, the enhancement disappeared in the 348-bp substrate-containing reaction (Fig. 5d, lane 10) when WT MutLα was replaced with an endonuclease-deficient MutLα, which contains an E705K substitution in the PMS2 subunit.^41^ These observations suggest that efficient mismatch removal in heteroduplexes, particularly those with a long distance between the mismatch and pre-existing strand break, depends on MutLα, which makes strand breaks 5′ to and near the mismatch (see below).

To directly visualize MutLα-catalyzed incisions, we performed Southern hybridization analysis using a 3′-nicked G–T heteroduplex and a ^32^P-labeled oligonucleotide probe (red bar) annealing 5′ to the mismatch on the nicked strand near the *Bgl*I site, so that the probe only detects incision products. We indeed observed multiple dominant incision species 5′ to the mismatch (Fig. 5e, see pink bracket and arrows) in the reaction carried out by the MMR-proficient HeLa nuclear extract (Fig. 5e, lane 1), but fewer such species in the MutLα-deficient H6 extract (Fig. 5e, lane 2). However, endonucleolytic cleavages were restored in H6 extracts when purified WT MutLα was added to these reactions (Fig. 5e, lanes 3 and 4); the restoration was not observed in H6 extract reactions supplemented with the E705K MutLα (Fig. 5e, lanes 5 and 6). The multiple incision species described above do not necessarily indicate that multiple cleavages occur on a single DNA molecule, as one molecule of the probe can only detect one DNA substrate molecule with a specific nick. Thus, each product represents a DNA substrate population with the same cleavage. Nevertheless, we cannot rule out that multiple incisions may occur on the same DNA substrate molecule. Relatively weak incisions 3′ to the mismatch were also observed (Fig. 5e, black arrows). Since these products are present in the H6 alone reaction, they may not be related to MutLα. Collectively, the data shown here strongly suggest that MutLα molecules are loaded onto DNA to conduct at least one incision 5′ to the mismatch on the newly synthesized DNA strand, followed by Exo1 recruitment to the nick to initiate mismatch removal. These results may also provide partial explanation for the delayed MMR for heteroduplexes with a longer distance between the mismatch and pre-existing nick. It is possible that multiple 5′ incisions occur between the two sites, so that Exo1 can be loaded to some or all of these nicks. This will result in a large DNA gap or a situation where DNA synthesis occurs in multiple locations within a few hundred of base pairs. Therefore, more time is likely needed to fill in a large DNA gap or to coordinate DNA synthesis in multiple locations. Future studies will define these possibilities.

MutL family proteins have been reported to act as sliding clamps during MMR initiation.^17,43^ To examine this possibility in functional MMR assay, we added LacI in the Southern hybridization assay described above. We found that LacI did not block incisions generated by MutLα within two LOS sites, but slightly altered the incision pattern (Fig. 5f, compare lanes 1 and 5 with lanes 2 and 6, respectively). This minor alteration was probably due to occupancy of the LOS-surrounding DNA sequences by LacI, which limited MutLα endonuclease activity. An obvious example is that the incision band at or near the LOS I site (see red asterisks in Fig. 5f) is present in reactions without LacI (lanes 1 and 5), but disappears in those with LacI (lanes 2 and 6). These data also indicate that like MutSα, sliding of MutLα is not required for MMR initiation.

It is worth mentioning that the most of incisions occurred 5′ to the mismatch (Fig. 5e, f). Based on the migration distances of these incision products and the known molecular size markers in Fig. 5f, we estimated the nucleotide distance of each of the 4 major incision bands (see green numbers) away from the mismatch. The estimation revealed that MutLα could make a 5′ nick that is 18 bp away from the mismatch. These results explain why MMR reactions containing heteroduplexes with various distances between the mismatch and pre-existing nick share essentially the same efficiency in mismatch removal (Fig. 5c). The data may also indicate that MutLα is involved in removing mispaired bases in Okazaki fragments, i.e., lagging strand MMR.

### MutLα interacts with DNA

**Fig. 6:**
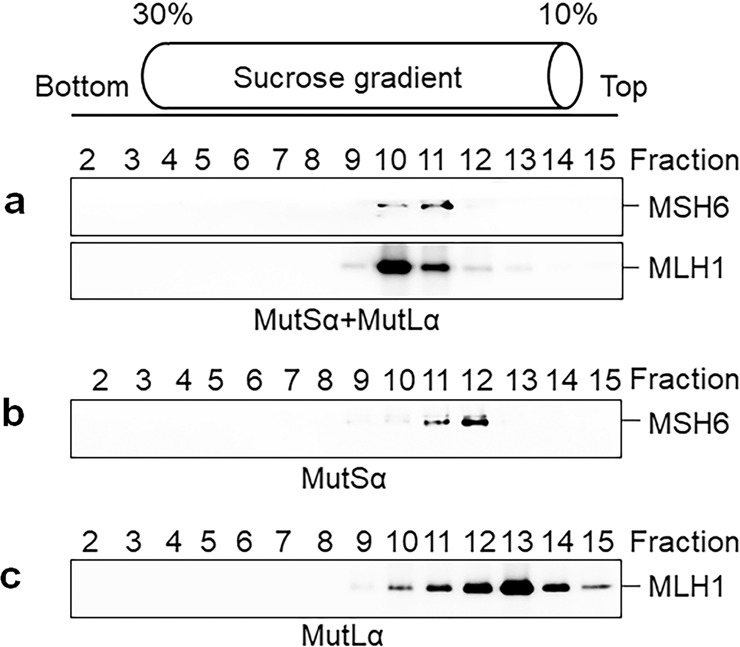
MutLα interacts with DNA. **a**–**c** Sucrose gradient centrifugation was performed to determine the molecular interactions between MutSα, MutLα, and heteroduplex DNA (**a**), MutSα and DNA (**b**), MutLα and DNA (**c**). Reaction mixtures were incubated on ice for 20 min, followed by centrifugation (16 h, 45,000 rpm, at 4 °C), fractionation (from the bottom to the top), electrophoresis, and western blotting.

We next asked how the MMR initiation complex efficiently communicates between mismatch and pre-existing strand break, which in turn triggers strand-specific incision near the mismatch by MutLα. Because an active MMR initiation complex contains multiple MutLα molecules (Fig. 4c), it is possible that MutLα, by interacting with MutSα, can be loaded to DNA in various places including the pre-existing nick. Thus, DNA bending by MutSα or the MutSα–MutLα complex can promote DNA looping through interactions between nick-located MutLα and mismatch-bound MutSα–MutLα complex, as proposed in the transactivation model.^19,20^ To test MutLα’s potential in this process, we performed sucrose density gradient sedimentation in the presence or absence of a 100-bp heteroduplex DNA. As expected, MutSα and MutLα form a ternary complex with DNA, as they co-sedimented in fractions 10 and 11 (Fig. 6a). Interestingly, we found that although MutSα (265 kD) is a larger molecule than MutLα (195 kD), a small fraction of the MutLα–DNA complex eluted one fraction earlier than the MutSα–DNA complex in sucrose gradient centrifugation (Fig. 6b vs c). This result suggests that more MutLα than MutSα molecules bind to DNA.

## Discussion

Canonical MMR involves mismatch recognition, strand discrimination, mismatch-provoked excision and incision, strand-specific mispair removal, and repair DNA synthesis. However, how MMR is initiated is not fully understood and the literature is highly controversial. The disagreement in current models of MMR initiation centers on whether MutS family proteins trigger mismatch-provoked excision by sliding from the mismatch to a pre-existing strand break or staying bound to the mismatch but communicating between the two sites through protein–protein interactions. We have provided convincing data to resolve this fundamental but controversial issue.

### The biological function of MutSα’s sliding activity in MMR

MutS family proteins are known to have ATP-dependent sliding activity^13–17^ and we showed that this activity is essential for MMR (Fig. 3f). The molecular switch model suggests that the sliding activity allows MutS family proteins to search for the strand discrimination signal by sliding from the mismatch to the pre-existing strand break. However, our results show that MutSα sliding is not involved in MMR initiation. First, when we blocked the MutSα sliding from the mismatch to the pre-existing strand break using LacI roadblocks, we still observed active excision at the strand break (Fig. 2e). Second, several sliding-deficient mutant MutSα proteins can trigger mismatch-provoked excision at the pre-existing strand break (Fig. 4a), which is consistent with a previous study showing that ATP binding-defective MutSα MSH2_G674A_·MSH6_WT_ or MSH2_WT_·MSH6_T1219D_ was able to initiate mismatch-provoked excision, but unable to complete excision in human nuclear extracts, although this was attributed to the failure of MutSα mutants to slide to the pre-existing strand break.^30^ However, these ATP binding-deficient MutSα mutants, as well as yeast ATP binding-deficient MutSα proteins Msh2_K694M_-Msh6_WT_ and Msh2_WT_-Msh6_K988M_, did not form a sliding clamp,^30,31^ consistent with the notion that MutS sliding is not required for triggering mismatch-provoked excision at the pre-existing nick. Finally, although MutSα alone slides away from the mismatch in the presence of ATP, MutSα forms a very stable and DNA-bound ternary initiation complex with MutLα in a manner that depends on ATP (Fig. 4c). This is in agreement with previous studies.^20,38^ Nevertheless, the *E. coli* MutS–MutL complex has been reported to slide slowly, but not slide off from the DNA.^23^ We suspect that the observed slow sliding away from the mismatch is consistent with our current finding that the MutS–MutL family complex yields the right of way to an exonuclease to remove the mismatch when the excision reaches the mismatch site.

In fact, native “roadblocks” are present at the replication fork. We found that ATP does not promote MutSα sliding in the EMSA assays as long as an MMR protein or DNA replication factor such as PCNA,^44^ RPA, or RFC is present in the reaction (Supplementary information, Fig. S3). In addition to these proteins, histone chaperones and nucleosome assembly factors are also present at replication forks.^29^ Thus, it is not easy for eukaryotic MutS family proteins to slide or travel a long distance along the DNA helix. Thus, the data shown here and documented in the literature,^30,31^ as well as physical barriers in vivo, do not support MutSα sliding in MMR initiation.

What role(s) does the sliding activity of MutS family proteins play in MMR? It has been postulated that MutS sliding along the DNA helix dissociates from homoduplex DNA and searches for a rare mismatch.^22^ Structural studies have shown that all MutS dimers (homodimer in prokaryotes and heterodimer in eukaryotes) contain two semi-closed channels, a mismatch recognition channel composed of the N-termini, and a second channel formed by the C-terminal residues of both subunits.^24–27^ The second channel’s properties, including stability, size, and electrostatic potential, facilitate nonspecific interactions with homoduplex DNA. Thus, when the mismatch recognition channel releases homoduplex DNA in the presence of ATP, the DNA can fall into the second channel, allowing MutS proteins to slide along the DNA helix and search for a mismatch.^22^ Although DNA metabolic factors and/or chromatin regulation proteins can block the sliding of MutS family proteins, the association of MutSα with individual nucleosomes in human cells^45^ indicates that there is no need for MutSα to travel a long distance. Nevertheless, this highly feasible MutS function has to be confirmed.

We have identified a hitherto unknown function of MutSα’s sliding activity, which is required for the last step of excision by yielding the right of way to Exo1. When conducting functional MMR assays using several ATP binding (hence sliding)-deficient MutSα proteins, we found that although these proteins can trigger both mismatch-provoked excision at the pre-existing strand break and incision near the mismatch (Fig. 4a, b), they block Exo1-catalyzed excision right at the mismatch site (Fig. 4a). This occurs because these proteins irreversibly bind to the mismatch (Fig. 3d), thereby preventing mismatch removal by Exo1. Since reactions with sliding-capable MutSα do not allow this type of excision intermediates to accumulate (Fig. 4a, b), sliding of MutSα or the MutSα–MutLα complex away from the mismatch is essential when the Exo1-catalyzed excision reaches the mismatch. Therefore, we conclude that the ATP binding-dependent sliding or DNA dissociation activity of MutS family proteins does not initiate mismatch-provoked excision at the pre-existing nick, but is essential for the last step of excision to finally remove the MutS-occupied mismatch.

### The role of MutLα in 5′-directed MMR

MMR can occur in either a 3′- or 5′-directed manner.^46^ In human cells, mismatch removal in 5′-directed MMR may rely on the Exo1-catalyzed excision all the way from the pre-existing strand break.^3,4,46^ However, we provide strong evidence that this may not be the case. Instead, we believe that as in 3′-directed MMR, 5′-directed repair is initiated by MutLα-generated incisions, and mismatch removal is achieved by Exo1-catalyzed excision at the MutLα-created nick that is 5′ closest to the mismatch. Our key evidence for this includes the following findings. First, despite an obvious delay in repair for heteroduplexes that have a longer distance between the mismatch and pre-existing nick than heteroduplexes that have a shorter distance between the two sites (Fig. 5a), there is no difference in the speed and efficiency of mismatch removal between these heteroduplexes (Fig. 5c, d). Second, effective mismatch removal depends on MutLα and its endonuclease activity (Fig. 5d). These observations strongly suggest that MutLα is required for 5′-directed MMR, and that the pre-existing strand break serves as the strand discrimination signal. This may apply to MMR in other organisms whose MutL homologue protein contains endonuclease activity.^41^ Our result is consistent with what is observed in *Xenopus* MMR, where repair DNA synthesis is similarly distributed around the mismatch among heteroduplexes with differential pre-existing strand breaks.^47^

However, previous in vitro reconstituted studies have shown that MutLα is dispensable in 5′-directed excision and repair.^35,48,49^ We think that the bypass of MutLα in these reactions is probably due to the 5′ strand break and the superactive 5′ → 3′ exonuclease Exo1. Thus, in the absence of the MutLα-generated strand breaks, Exo1 is loaded to the pre-existing break to conduct excision until the mispaired base is removed. Given that the Exo1-catalyzed excision can be easily blocked by LacI (Figs. 2 and 4), Exo1 may not be able to excise DNA in vivo as it does in vitro. This is because numerous DNA metabolic proteins and chromatin structural factors are present at replication forks. These factors can act as roadblocks to inhibit Exo1 excision. Thus, like MutSα sliding, Exo1 excision is probably restrained to a short distance, however, the MutLα-created nick that is 5′ to and near the mismatch is the ideal site for Exo1 to initiate mismatch removal. Therefore, we believe that the endonuclease activity of MutLα is essential for 5′-directed MMR in vivo.

### Working model

Ruling out the involvement of MutS sliding activity in initiating MMR excision raises the question of how mismatch binding by the MutS family proteins identifies the strand discrimination signal to activate mismatch-provoked excision/incision. Previous studies have linked this to another essential MMR initiation factor, MutL in *E. coli* and its eukaryotic homologue MutLα. Hombauer et al.^21^ observed that although yeast MLH1-PMS1 (MutLα in yeast) relies on MutSα to form nuclear foci, it rarely colocalizes with MutSα. Thus, Hombauer et al.^21^ postulated that mismatch-bound MutSα recruits multiple molecules of MutLα, which, in turn, loads Exo1 to DNA to remove mismatches. However, how MutLα communicates with strand discrimination signal is not fully understood. According to the transactivation model (Fig. 1c), the MMR initiation complex remains bound to the mismatch, and MutL family proteins activate downstream activities at the strand discrimination signal via DNA bending/looping.^19,20,22,24^

**Fig. 7:**
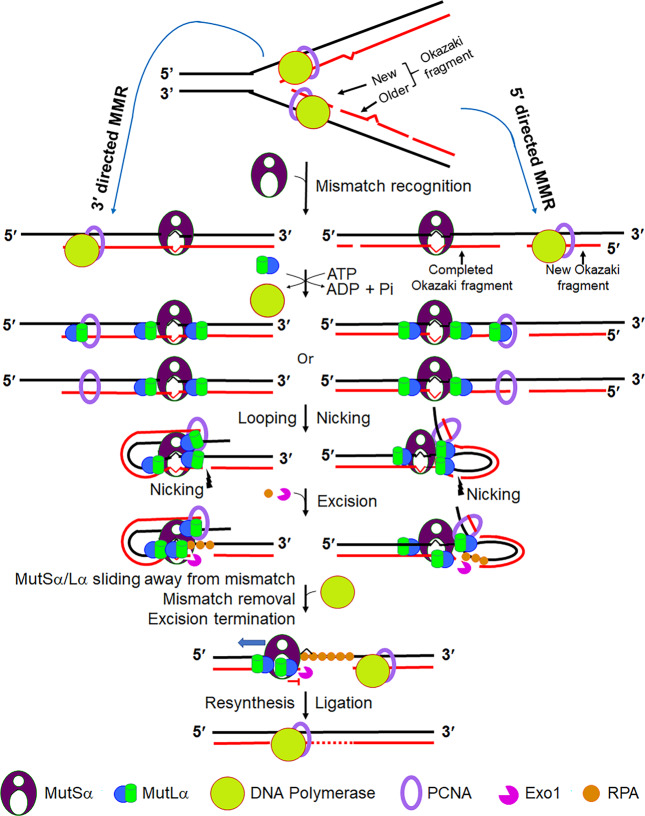
Model of the human MMR process. Misincorporation can occur in either leading or lagging strand DNA synthesis, and these mispairs can be corrected in a 3′- or 5′-directed manner. Mismatch-bound MutSα recruits MutLα to DNA to form a stable initiation complex, in an ATP-dependent manner. In this ternary complex, mismatch-bound MutSα is flanked by MutLα molecules, and MutLα–MutLα or PCNA–MutLα/MutSα interactions bring mismatch and strand break to proximity, which simplifies the communication between the two sites. MutLα then makes a nick 5′ to the mismatch on the nicked strand. Exo1 is recruited by MutLα to the nick and conducts 5′ → 3′ excision. Once the Exo1-catalyzed excision reaches the mismatch, MutSα or the MutSα–MutLα complex slides away from the mismatch, yielding the right of way to Exo1 for mismatch removal. The excision is terminated by the interactions between MutLα and Exo1. The DNA gap is filled by DNA polymerase *δ* in concerted reactions with PCNA and RPA, and the nick is ligated by ligase I. This model applies to both 3′ nick-directed (left panel) and 5′ nick-directed (right panel) MMR.

Our data presented here support both transactivation and multi-MLH loading models. First, we showed that an active MMR initiation complex contains more than one MutLα (Fig. 4c); failure to assemble the initiation complex with multiple molecules of MutLα leads to defective MMR initiation (Fig. 4a, lanes 8 and 9) and overall repair (Fig. 3f). Second, MutLα makes at least one incision on the nicked DNA strand 5′ to the mismatch, regardless of 3′- or 5′-directed MMR (Fig. 5). Third, MutLα by itself appears to interact with DNA and form a multimer complex (Fig. 6), possibly through its long flexible linker arms between the N-terminal ATPase and C-terminal dimerization domains.^50,51^ Recent studies have revealed that MutLα molecules interact with each other and compact mismatched DNA via flexible arms.^52,53^ PCNA is loaded directionally according to the strand polarity^54,55^ and directs MutLα endonuclease cleavage.^41^ Because both MutLα subunits, MLH1 and PMS2, interact with PCNA,^56,57^ and because MutLα foci rarely colocalize with MutSα foci,^21^ MutLα may be directly recruited to the DNA elongation site (i.e., free 3′ end) by interacting with PCNA in a manner that depends on a mismatch but not necessarily on MutSα. Thus, MutLα molecules that bind at the mismatch and pre-existing strand break can loop DNA as proposed in the transactivation model^22,50^ (Fig. 1c). Misincorporation may also trigger PCNA to dissociate from DNA polymerases and interact with MutSα and MutLα, turning on its MMR function. Thus, loop formation between the mismatch and strand discrimination signal can be promoted through the interactions between PCNA located at or near the 3′ end of the elongation site and MutLα or the MutSα–MutLα complex at the mismatch (Fig. 7). These possibilities need to be confirmed in future studies.

An MMR model that summarizes our results along with published studies is shown in Fig. 7. A misincorporated nucleotide occurring during DNA replication, either on the leading or lagging strand, is recognized by MutSα, which recruits multiple molecules of MutLα in an ATP-dependent manner to form a mismatch-bound MutSα–MutLα complex that initiates MMR. MutLα can also be recruited to the site of DNA synthesis through its interaction with PCNA. Physical interactions between MutLα (or PCNA) and MutLα at a strand break (or free DNA end) bring mismatch and strand break to proximity, forming a DNA loop. This allows effective communication between the two sites by providing both strand signal and targeted area for incision/excision. MutLα then makes incisions 5′ near the mismatch on the newly synthesized strand (Fig. 5e, f). The nicked DNA molecule is ready to be processed further by either unidentified nucleases as described previously,^58,59^ or Exo1. In the latter case, which is the focus of this study, Exo1 is recruited by MutLα to act at the MutLα-generated nick. Once the Exo1-catalyzed excision reaches the MutSα- or MutSα–MutLα-bound mismatch, MutSα or the MutSα–MutLα complex slides away from the heterology, yielding the right of way to Exo1 so that the mispaired base can be removed. Upon mismatch removal, the Exo1-catalyzed excision is terminated by MutLα through the physical interaction between these two proteins^35^ because disrupting the MutLα–Exo1 interaction leads to uncontrolled Exo1 excision.^9^ DNA gaps are filled by DNA polymerase δ in coordination with other DNA synthesis factors including RFC, PCNA, and RPA, and the repair is completed by ligase I-catalyzed DNA ligation.

## Materials and methods

### Cell culture and nuclear extract preparation

Cell lines HeLa and NALM-6 were grown in Roswell Park Memorial Institute 1640 with 5% (vol/vol) fetal bovine serum (FBS) and 4 mM L-glutamine at 37 °C in a humidified atmosphere with 5% (vol/vol) CO_2_. The HCT116 line was cultured in McCoy’s 5 A under the same conditions. Nuclear extracts derived from these cells were prepared as described.^36^

### Heteroduplex preparation

The DNA heteroduplexes used in this study were 7.1-kb double-stranded circular molecules (Fig. 2a) that contained a G–T mismatch and a strand break 242 bp 5′ to the mismatch (5′ substrate) or a strand break 181 bp 3′ to the mispair (3′ substrate). The G–T mismatch is located within the overlapping recognition site of two restriction enzymes, *Nsi*I and *Xho*I. The heteroduplexes were derived from bacterial phages, M13mp18-UKY1 and M13mp18-UKY2,^33^ and contain a LOS sequence at either side of the mismatch. The LOS I sequence (5′-AATTGTGAGCGGATAACAATT-3′) was cloned into the *Hind*III site, and is 67 bp 5′ to the mismatch; the LOS II, 62 bp 3′ to the mismatch, is the native lac operon in the bacterial phage. The distance between two LOS sites is 130 bp.

### Protein purification and MMR assays

Of the proteins used in this study, LacI, PCNA, and RPA were expressed in *E. coli*, and MutSα, MutLα, and Exo1 were expressed in insect cells; all proteins were purified to reach homogeneity as previously described.^35,60^ The *LacI* plasmid (pBR322) was a gift from Dr. Kathleen S. Matthews (Rice University, USA).

MMR assays were performed in 20-μL reactions containing 25 fmol mismatched DNA (Fig. 2a), 50 μg nuclear extracts or reconstituted MMR system (see below), 10 mM Tris-HCl (pH 7.5), 5 mM MgCl_2_, 1.5 mM ATP, 0.1 mM dNTPs, and 110 mM KCl. Unless otherwise specified, 400 fmol MutSα and 280 fmol MutLα, 800 fmol RPA, and 300 fmol PCNA homotrimer, and 15 fmol Exo1 were used in reconstituted MMR reactions. The reaction mixtures were assembled on ice, incubated at 37 °C for 15 min, and terminated by proteinase K digestion. Repair was scored by restriction enzyme digestion and visualized after gel electrophoresis as described.^35^

Mismatch removal assays were conducted essentially in the same way as the repair assay, except for dNTPs being omitted in the nuclear extract reaction. Mismatch removal was scored by the conversion of double-stranded substrates to gapped molecules using restriction enzymes *Nhe*I and *Cla*I as described.^33^ The mismatch-removed molecules are resistant to *Nhe*I (Fig. 5b).

Mismatch-provoked incision/excision assays using nuclear extracts were conducted by omitting dNTPs from the standard MMR assay, which blocks repair DNA synthesis. For reconstituted MMR reactions, minimum required proteins MutSα, MutLα, RPA, Exo1, and PCNA were used. Unless mentioned otherwise, all reactions were assembled on ice, and then incubated at 37 °C for 15 min. For reactions containing LacI, the protein (160 nM in tetramer) was pre-incubated with DNA substrates on ice for 10 min before adding other components. DNA samples were digested with *Pst*I and *Bgl*I and fractionated by a 6% (wt/vol) denaturing polyacrylamide gel, followed by Southern blot analysis using a ^32^P-labeled oligonucleotide probe complementary to the nicked strand either near the *Bgl*I site or the *Pst*I site. Excision/incision products were visualized by a phosphor imager.

### EMSA

Unless otherwise specified, EMSA was performed to determine the interactions between LacI and LOS, MutSα and heteroduplex DNA, and MutSα–MutLα and heteroduplex DNA, using a ^32^P-labeled 282-bp heteroduplex flanked by a LOS sequence at either side of the mismatch. EMSA assays were conducted in 20-µL reactions containing 0.1 pmol ^32^P-labeled heteroduplex DNA, 1 pmol of non-labeled homoduplex DNA, 0.6 pmol LacI tetramer (if present), 0.4 pmol of MutSα, 10 mM HEPES (pH 7.5), 200 µg/mL BSA, 5 mM MgCl_2_, 1 mM DTT, 110 mM KCl, and 2 mM ATP (if present). After 20-min incubation on ice, 5 μL of 50% (w/v) sucrose was added, and the samples were subjected to electrophoresis at room temperature through 4% non-denaturing polyacrylamide in 6.7 mM Tris-acetate (pH 7.5) and 1 mM EDTA as described previously.^61^

### ATP binding and ATPase assay

MutSα ATP binding and ATPase assays were performed in 20-μL reactions containing 25 mM HEPES (pH 7.5), 120 mM KCl, 5 mM MgCl_2_, 1 mM DTT, 100 nM MutSα, and 0.2 μCi [γ-^32^P]-ATP, as previously described.^62^ In the ATP binding assay, 25 fmol heteroduplex DNA was used when specified in the reactions. Samples were incubated on ice for 10 min, followed by 10 min of UV cross-linking (Stratalinker). Products were resolved in 8% SDS-PAGE. In the ATPase assay, reactions were incubated in the presence of 25 fmol DNA for 10 min at 37 °C, and fractionated through a 20% denaturing polyacrylamide gel. Radiolabeled products were detected by a Typhoon PhosphoImager and quantified by ImageJ.

### Sucrose gradient centrifugation

Sucrose gradient centrifugation was performed to determine the molecular interactions between MutSα, MutLα, and heteroduplex DNA. Reactions were assembled in 20-µL reactions containing 40 pmol of each indicated protein, 2 pmol of mismatched DNA (a 100-bp G–T mismatch-containing oligonucleotide duplex), 10 mM Tris-HCl (pH 7.5), 5 mM MgCl_2_, 1.5 mM ATP, and 110 mM KCl. Reaction mixtures were incubated on ice for 20 min, and then loaded onto the top of 5-mL swinging buckets containing a sucrose gradient of 10%–30%. The samples were centrifuged at a speed of 45,000 rpm at 4 °C for 16 h, followed by fractionation (from the bottom to top), SDS-PAGE, and western blotting.

## Supplementary information

Supplementary information, Figure S1

Supplementary information, Figure S2

Supplementary information, Figure S3
