# Supplementary material for: Mispair-bound human MutS–MutL complex triggers DNA incisions and activates mismatch repair
Source: Cell Res. 2021 Jan 28;31(5):542–53. doi: 10.1038/s41422-021-00468-y (PMC8089094; doi:10.1038/s41422-021-00468-y)
Supplement: Supplementary file 1 — Supplementary information, Figure S1 [file 41422_2021_468_MOESM1_ESM.pdf]

Supplementary information, Figure S1

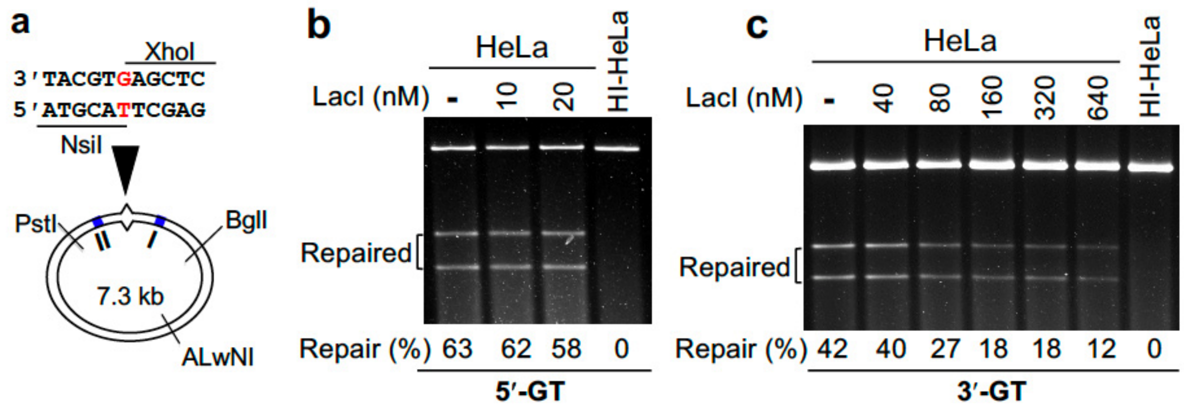

Fig. S1 LacI-roadblocks cannot completely inhibit MMR in vitro. **a** Mismatched DNA substrate. The curcular DNA substrate contains a G-T mismatch located in the overlapping recognition sequence of XhoI and NsiI, so that both enzymes cannot cleave the DNA. However, upon mismatch removal and subsequent DNA repair synthesis, restriction recognition sequence is restored to one of the enzymes, which is used to score for repair. The substrate also contain a LacI binding sequence (blue boxes I and II) either side of the mismatch, and a strand break either 242-bp 5' (at the BglI site) or 181-bp 3' (at the PstI site) to the mismatch. **b** MMR assays performed using 5'-nicked DNA substrate (5' G-T). **c** MMR assays using 3'-nicked substrate (3' G-T). In vitro MMR assays were perfromed in 20-µL reactions containing 50 µg of HeLa nuclear extracts or heat-inactivated (HI) HeLa extracts as indicated, 25 fmol (1.25 nM) of 5' or 3' G-T substrate, 10 mM Tris-HCl (pH 7.6), 5 mM $MgCl_2$, 1.5 mM ATP, 110 mM KCl, 0.1 mM each of the four dNTPs and the indicated amount of purified LacI. The reaction mixtures were incubated at 37 °C for 15 min, and DNA samples were recovered by phenol extraction and ethanol precipitation, followed by double-digestion with ALwNI and NsiI (the score enzyme) and agarose gel electrophoresis. DNA repair products were visualized by UV-illumination in the presence of ethidium bromide.
