# Supplementary material for: Mispair-bound human MutS–MutL complex triggers DNA incisions and activates mismatch repair
Source: Cell Res. 2021 Jan 28;31(5):542–53. doi: 10.1038/s41422-021-00468-y (PMC8089094; doi:10.1038/s41422-021-00468-y)
Supplement: Supplementary file 2 — Supplementary information, Figure S2 [file 41422_2021_468_MOESM2_ESM.pdf]

Supplementary information, Figure S2

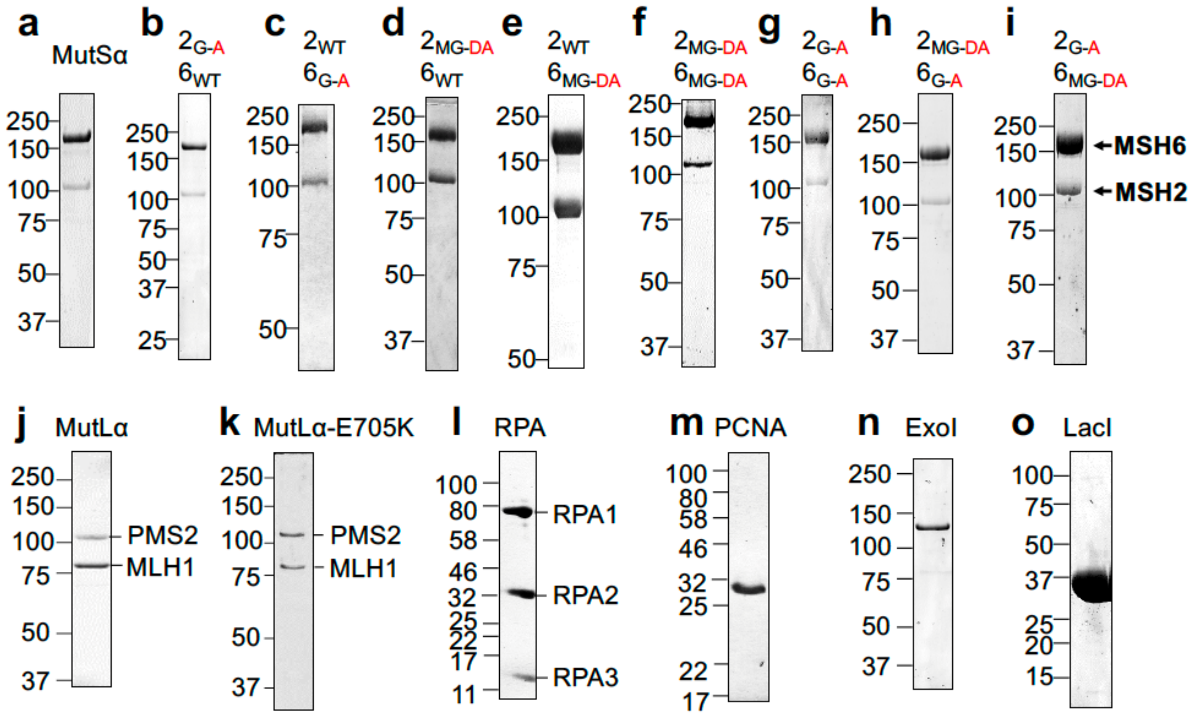

Fig. S2 SDS-PAGE of recombinant MMR proteins used in this study. a-i WT or mutant MutSα, as indicated. **j** MutLα. **k** MutLα(E705K). **l** RPA. **m** PCNA. **n** Exo1. **o** LacI. All proteins, except RPA, PCNA and LacI, which were expressed in *E. coli* cells, were expressed in insect cells through the baculovirus system.
