# Supplementary material for: Mispair-bound human MutS–MutL complex triggers DNA incisions and activates mismatch repair
Source: Cell Res. 2021 Jan 28;31(5):542–53. doi: 10.1038/s41422-021-00468-y (PMC8089094; doi:10.1038/s41422-021-00468-y)
Supplement: Supplementary file 3 — Supplementary information, Figure S3 [file 41422_2021_468_MOESM3_ESM.pdf]

Supplementary information, Figure S3

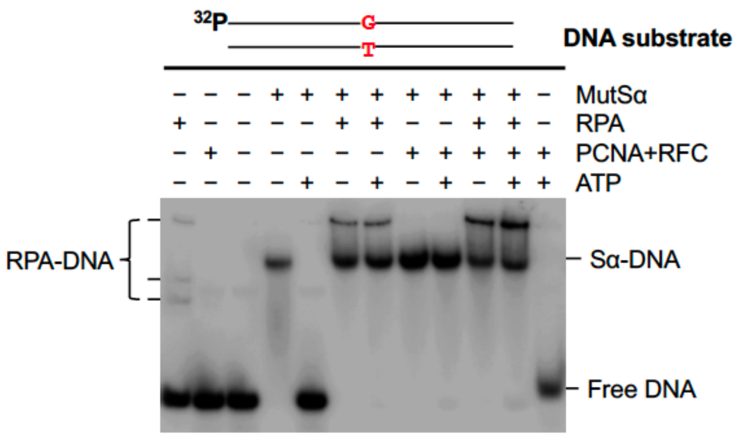

Fig. S3 ATP-triggered MutSα sliding is inhibited in the presence of DNA replication proteins. Electrophoretic mobility shift assay (EMSA) was performed to determine the interactions between MutSα and heteroduplex DNA, and MutSα-DNA and other MMR proteins, using a ^{32}P-labeled 100-bp G-T-containing heteroduplex. Individual reactions were assembled, as indicated, and incubated on ice for 20 min with 10 mM HEPES (pH 7.5), 200 µg/mL BSA, 10 mM $MgCl_2$, 5 mM DTT, 110 mM KCl. The reactions were terminated by adding 5 µL of 50% (w/v) sucrose and analyzed by 4% non-denaturing polyacrylamide gel. DNA and its protein complexes were visualized using a phosphor imager. The results show that ATP-stimulated MutSα sliding is inhibited when reactions contain RPA, PCNA or RFC. Interestingly, despite that these proteins enhance MutSα-DNA interaction, no supershifted complexes were identified, consistent with a previous study, where PCNA enhances MutSα's DNA-binding activity, but does not form a supershifted complex with MutSα (Flores-Rozas, H., Clark, D., and Kolodner, R.D. Nat Genet ***26***, 375-378, 2000).
